# Snyder and Champness Molecular Genetics of Bacteria, 5th Edition

**DOI:** 10.3201/eid2801.211628

**Published:** 2022-01

**Authors:** Rachel A. Moore, Christopher E. Carr

**Affiliations:** Georgia Institute of Technology, Atlanta, Georgia, USA

**Keywords:** microbiology, genomics, genetic models, E. coli, DNA, bacteria

To a molecular biologist, a textbook so heavily focused on the classical genetics of model systems such as *Escherichia coli* and *Bacillus subtilis* might, at first glance, come across as “been there, done that.” And yet, looking closely at a topic well studied is often a route to new knowledge and can allow us to test heretofore unrecognized assumptions in new ways. Such is the importance of condensing our ever-expanding knowledge into one thorough and digestible text.

The history of Snyder and Champness Molecular Genetics of Bacteria goes back more than 24 years (Figure). As a tribute to its original authors, Larry Snyder and Wendy Champness, the book was renamed for this fifth edition. The current authors, Tina M. Henkin and Joseph E. Peters, have skillfully updated the classic text to address the latest advances in scientific knowledge and technology. The text also contains many useful features to summarize main points, lists of suggested reading at the end of chapters, and full-color illustrations that will appeal to anyone intent on teaching or understanding the inner workings of bacterial molecular genetics.

**Figure:**
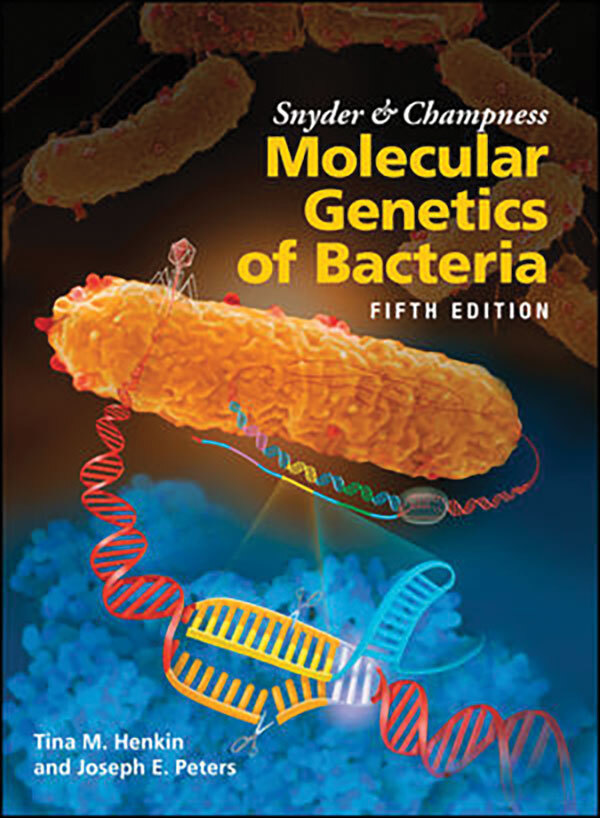
Snyder and Champness Molecular Genetics of Bacteria, 5th Edition

Reading through each chapter, the reader can follow along with a sort of classical genetics take on molecular biology. That is, the book has a logical flow through 13 chapters, beginning with DNA structure, replication, and gene expression and ending at DNA repair, regulation, and genomic analysis. The introduction and first chapter, The Bacterial Chromosome, are designed to provide a basic background in genetics. They include historical perspectives and information on the molecular tree of life and the basic building blocks of DNA. The ensuing chapters build upon that introductory material and include more specific examples primarily centered around the most well-studied and understood bacteria.

Results from peer-reviewed scientific articles are woven in throughout the book. Examples include the carbon storage regulator global regulatory network in *Escherichia coli*, *lacZ* mutations producing blue pigment, and the stages of *Bacillus subtilis* sporulation. The authors subtly introduce the reader to primary sources, in addition to bridging the gap between research and the classroom.

The new final chapter, Genomes and Genomic Analysis, is a welcome addition that provides background on laboratory techniques. The sections on sequencing methods could use more detail on new strategies (e.g., the diversity of short-read and long-read approaches to sequencing and their relative advantages and limitations). In addition, and of lesser importance, the Sanger sequencing “cart” is, if you will, presented before the PCR “horse.” Perhaps the next edition can delve deeper into these ever-evolving technologies.

Overall, the book contains material perfect for teaching a university-level course on bacterial molecular genetics. It also includes information for students and professionals (e.g., current or aspiring synthetic biologists) seeking to update their knowledge to the current state of the art of bacterial genetics.

